# Medical Isolation and Solitary Confinement: Balancing Health and Humanity in US Jails and Prisons During COVID-19

**DOI:** 10.1007/s11606-020-05968-y

**Published:** 2020-07-06

**Authors:** David H. Cloud, Cyrus Ahalt, Dallas Augustine, David Sears, Brie Williams

**Affiliations:** 1 Division of Geriatrics, Department of Medicine, School of Medicine, University of California, San Francisco, San Francisco, CA USA; 2 Division of Infectious Diseases, Department of Medicine, School of Medicine, University of California, San Francisco, San Francisco, CA USA

## Abstract

In the face of the continually worsening COVID-19 pandemic, jails and prisons have become the greatest vectors of community transmission and are a point of heightened crisis and fear within the global crisis. Critical public health tools to mitigate the spread of COVID-19 are medical isolation and quarantine, but use of these tools is complicated in prisons and jails where decades of overuse of punitive solitary confinement is the norm. This has resulted in advocates denouncing the use of any form of isolation and attorneys litigating to end its use. It is essential to clarify the critical differences between punitive solitary confinement and the ethical use of medical isolation and quarantine during a pandemic. By doing so, then all those invested in stopping the spread of COVID-19 in prisons can work together to integrate medically sound, humane forms of medical isolation and quarantine that follow community standards of care rather than punitive forms of solitary confinement to manage COVID-19.

The nation’s poor preparation and slow response to the risks posed by COVID-19 have been compounded in jails and prisons, many of which are now reporting high numbers of infections among incarcerated people and staff. Evidence so far indicates that correctional facilities, including jails in New York City and Chicago and prisons in Ohio, have the highest rates of confirmed cases of COVID-19 of any setting.^1^ As the COVID-19 pandemic sweeps through an alarming number of US jails and prisons, guidance from the medical community on the ethical and humane use of medical isolation and quarantine in these settings is urgently needed. While social distancing is critical to slowing the spread of disease, it is exceedingly challenging in the unique settings of prisons and jails. Consequently, some correctional systems are employing isolation in ways that are haphazard or inhumane and will, in turn, undermine their public health intentions. Recently, some prison reform advocates and litigators working on behalf of incarcerated people have called for a prohibition against the use of isolation to combat COVID-19, out of concern that recent strides made toward ending solitary confinement in the USA are being put in jeopardy.^2, 3^ This tension, left unresolved, could rapidly result in a health and humanitarian crisis affecting the residents, employees, and surrounding communities of our nation’s over 5000 places of detention.

## RESPONDING TO COVID-19 IN CORRECTIONS: UNIQUE CHALLENGES TO MEDICAL ISOLATION AND QUARANTINE

From a public health perspective, the concerns of both correctional systems and prison reform advocates are valid. Solitary confinement is a punishment, in widespread use in US correctional facilities, despite a wealth of evidence that it contributes to excess morbidity and mortality among currently and formerly incarcerated people.^4–7^ The hallmarks of solitary confinement—social isolation, physical idleness, and sensory deprivation—lead to immense psychological suffering and lasting trauma, and too often result in self-harm, violence, and suicide, even after only relatively short periods of time.^4, 8^ Following decades of litigation and advocacy, an increasing number of state correctional systems have adopted reforms focused on curbing and eventually eliminating solitary confinement.^9^ Even states with high rates of incarceration and greater proportions of their prisoner population in solitary confinement, such as Louisiana, have made notable progress in this area of punishment.^10^ Many advocates fear that use of isolation to curb transmission of COVID-19 in correctional facilities will complicate the emerging crisis, as incarcerated people become reluctant to report symptoms for fear of being moved to solitary confinement, those who do report symptoms will be forced to endure an experience known to cause psychological and physical harm, and system-wide unrest will be triggered in institutions where fears about being placed in medical isolation could run rampant.

Yet, quarantine and medical isolation in response to COVID-19 are necessary to halt the spread of infection; without them, containment of disease transmission will be exceedingly difficult if not impossible, posing significant health risks to incarcerated people, correctional healthcare providers, security staff, and the families and communities to which workers return at the end of each shift. Clearly defining and instituting the ethical and humane application of well-established medical isolation and quarantine practices in the community is critical for curbing transmission in these highly hazardous environments and protecting the dignity of incarcerated people.

## EFFECTIVE MEDICAL ISOLATION AND QUARANTINE REQUIRES SIGNIFICANT POPULATION REDUCTION AND A NEW APPROACH TO POPULATION MANAGEMENT

Most US jails and prisons continue to operate at or above their designed capacity and are chronically understaffed in both security and healthcare services. Overcrowding renders social distancing efforts unlikely to be successful once COVID-19 is introduced into a US jail or prison. As a result, any effective and ethical medical isolation and quarantine program in US jails and prisons must be preceded by the immediate release of as many people as possible from jails and prisons to ensure that adequate physical space and medical staff are available for the population that remains.^11^

Unfortunately, the numbers of releases in most jurisdictions to date have been relatively small and woefully insufficient. Given the grave health risks that COVID-19 pose to correctional institutions and their surrounding communities, corrections officials and advocates for incarcerated people and their families should find common cause in persuading governors, legislators, and the public that rapid decarceration, including of the sentenced population, is necessary and can be done safely.^12^ Population reduction efforts should only be deemed adequate once every facility can safely accommodate complete social distancing by newly organized cohorts of incarcerated people; these cohorts should be sized with some consideration of medical care capacity in the facility and surrounding community and based on the family unit in communities under “stay home” orders. Facilities must also have the capacity to clean shared spaces (including eating, recreating, and bathing areas) regularly so that these cohorts can serve as a first-line defense against the broader spread of infection already evidenced in some jails and prisons.

Once populations are reduced to a manageable level, temporary and humane medical isolation and quarantine for those exposed to or infected with COVID-19 must be used to stem transmission of the virus and mitigate the worst outcomes associated with a surge in cases. In this context, it is critical to clearly distinguish between solitary confinement, medical isolation, and quarantine so that leaders, practitioners, and advocates are working to support appropriate medical responses to outbreaks in correctional facilities.

## MEDICAL ISOLATION AND QUARANTINE SHOULD BEAR LITTLE RESEMBLANCE TO SOLITARY CONFINEMENT

“Medical isolation” and “quarantine” procedures are substantively different from “solitary confinement” (Table 1). However, critical misperceptions persist inside and outside correctional facilities about what these procedures should be and how they affect the people living and working in prisons and jails.

**Table 1:** Comparing Solitary Confinement, Medical Isolation, and Quarantine

	Solitary confinement	Quarantine	Medical isolation
Mechanism	Separation of people from population as a means of punishment	Separation of people exposed to contagious disease from population	Separation of people with a contagious disease from population
Purpose	Punitive	Reduce spread of disease	Reduce spread of disease
Duration	Determined by custody	Determined by medical staff, until incubation period passes or status is changed to medical isolation if patient develops disease	Determined by medical staff, until person is deemed no longer contagious

The only commonality that solitary confinement should share with quarantine and medical isolation is a physical separation from other people. In fact, those in medical isolation may be housed together with others who also have COVID-19. This means that people in quarantine or medical isolation should have enhanced access to resources that can make their separation psychologically bearable—for example, television, tablets, radio, reading materials, and means of communicating with loved ones—since they are enduring isolation for the greater good, not for punishment. They should have easy access to medical and mental health professionals, and daily updates from healthcare staff as to why separation is necessary and how long they can expect it to last. Corrections officials should make additional efforts to communicate with and show compassion for people in their custody who are scared and feeling unwell in quarantine or medical isolation.^13^ Some simple ideas include distributing cell phones, tablets, televisions, gaming consoles, and other equipment that people in medical isolation, quarantine, or sheltering-in place in the community may be using to cope with the anxiety of isolation. Healthcare providers working outside corrections could offer telehealth consultations with patients via tablets and other HIPAA-compliant digital platforms. If corrections systems lack these resources, public health agencies, non-profit organizations, advocates, faith-based entities, and philanthropies should mobilize to assist in providing them.

In many correctional facilities, the only available spaces for implementing quarantine or medical isolation are those typically used for punishing people with solitary confinement. This is because these units have single cells with solid cell doors and are removed from communal living areas. Repurposing solitary confinement units for medical purposes, however, runs the risk of corrections officials falling back on policies that subject people to living conditions known to harm their health. It is imperative that if these units are used to contain the COVID-19 epidemic, there must be accompanying communication from medical and correctional staff to the wider population and clear examples of how housing in these units will differ from “run of the mill” solitary confinement. Similarly, any housing used as part of a medical response must be medically appropriate with, for example, proper ventilation and adequate sanitation. Given the high rates of comorbid conditions in correctional settings, corrections and public health officials should ensure that people undergoing quarantine do so in reasonable proximity to urgent care.

Additionally, solitary confinement is often used for extended or even indeterminate periods of time, with release back to general population housing at the discretion of correctional officers. In stark contrast, quarantine and medical isolation are temporary procedures that should be overseen by medical professionals. CDC guidelines for discontinuing quarantine and medical isolation should govern decisions in jails and prisons, just as they do in the community.^14^ Community standard length of time for quarantine and medical isolation, on average about 14 days, aligns closely with (and do not exceed) the United Nation’s Standard Minimum Rules for the Treatment of Prisoners (the “Nelson Mandela Rules”) that define punitive use of solitary confinement for longer than 15 days as “torture”^15^ (Table 2).

**Table 2:**
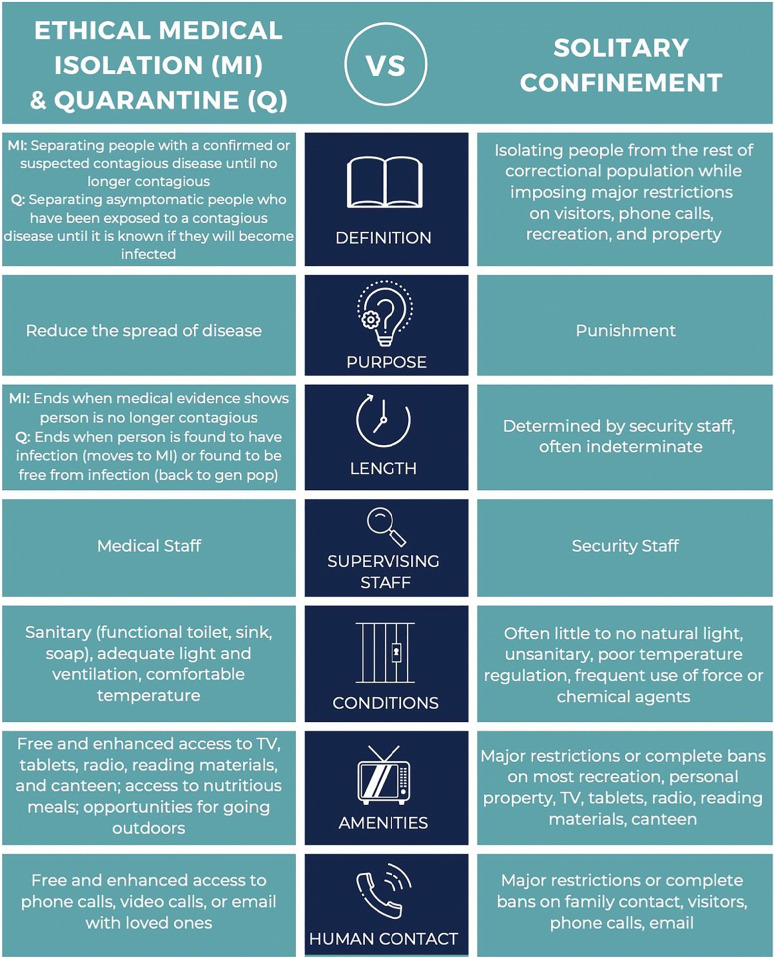
Characteristics of Solitary Confinement and Medical Isolation

## TESTING CAPACITY IS CRITICAL TO THE ETHICAL USE OF MEDICAL ISOLATION AND QUARANTINE

Implementing time-restrictions for quarantine and medical isolation in any setting hinges on having sufficient capacity for testing patients for COVID-19. While some facilities have increased testing efforts for residents and staff, others have not. Without system-wide testing, disease prevention strategies will fail and result in collateral harms.^16^ If healthcare staff lack knowledge of infected residents and staff, then they cannot make informed decisions about quarantine or medical isolation. This lack of testing will increase anxiety and stress among residents and staff in the correctional setting and could lead to widespread and indiscriminate solitary confinement, which would compound unrest and mistrust. Therefore, it is critical for corrections, public health, and medical officials to allocate resources necessary for robust COVID-19 testing kits for people living and working in jails and prisons.

## BALANCING HEALTH AND HUMANITY IN THE CONTEXT OF COVID-19

The use of punitive isolation during the COVID-19 epidemic—including indeterminate system-wide facility lockdowns where people cannot communicate with their families, exercise outside, participate in programming, or interact with healthcare professionals—will deter people from reporting symptoms, in turn threatening the health of all those who work in jails and prisons. Because the correctional workforce returns home at the end of each shift, outbreaks in correctional facilities threaten the health of those in surrounding communities. With the COVID-19 crisis in jails and prisons worsening daily, many under-resourced correctional health systems are already overwhelmed by this crisis. This is not business as usual in prison reform. Elected officials, correctional leaders, advocates, litigators, family members, and others concerned about people behind bars must work together for immediate population reduction in our jails and prisons and to secure the needed medical supplies, testing kits, staffing support, and other vital resources for those who remain incarcerated and the staff supervising and caring for them.

Collective action among uncomfortable allies is a challenging but necessary strategy for minimizing the harms of this pandemic. But to meet the demands of this rapidly unfolding crisis, now is the time to put health first in correctional policy and practice, release *all* people who can be safely released to the community, and ensure that medical isolation and quarantine procedures follow community standards of care and are not, in reality, solitary confinement by another name.
